# Supplementary material for: A Likelihood Approach for Real-Time Calibration of Stochastic Compartmental Epidemic Models
Source: PLoS Comput Biol. 2017 Jan 17;13(1):e1005257. doi: 10.1371/journal.pcbi.1005257 (PMC5240920; doi:10.1371/journal.pcbi.1005257)
Supplement: S1 File — (TAR.GZ) [file pcbi.1005257.s014.tar.gz › HSPH_Online-SI-Revision/Additions/IPoi10/n100-extreme-SEITR_noisy/n100-extreme-SEITR_noisy_BENCH_table-8.pdf]

1	R0	{1.38654, 9.85565}	{1.12441, 1.83859}	{{1.12171, 1.78492}, {1.12171, 1.78492}}	{18.9056, 81.3448}	inf	369.	497.067	{224., 660.}	27.1401	0.132274	newly	644.29	1918.93	{913., 2520.}	200.223	0.454091	Reff	1.36736	1.02483	{0.931591, 1.09255}	25.0503	ar	92379.	21278.1	{17885., 24447.}	76.9665
2	R0	{2.36629, 12.2029}	{1.44746, 17.6177}	{{1.30812, 12.6353}, {1.67375, 19.7105}}	{38.8298, 47.8187}	inf	10.	2.59649	{0., 5.}	81.9549	0.951971	newly	20.	1.17544	{0., 3.}	94.1228	1.57187	Reff	2.3656	1.48816	{1.3435, 1.75635}	37.0919	ar	99022.	27258.3	{1., 67327.}	72.4725
3	R0	{2.32253, 12.836}	{1.44884, 11.8527}	{{1.0158, 8.09734}, {2.21449, 18.33}}	{39.4481, 30.7228}	inf	18.	1.92	{0., 5.}	92.8889	1.60001	newly	26.	1.29	{0., 4.}	95.0385	1.77736	Reff	2.32139	1.44879	{1.01576, 2.2144}	39.4275	ar	98997.	18764.	{1., 71622.}	81.0459
4	R0	{1.79075, 11.2058}	{1.37684, 13.9202}	{{1.0158, 8.09734}, {1.85141, 19.5412}}	{26.2195, 41.0138}	inf	68.	5.23214	{0., 14.}	94.606	1.6869	newly	88.	3.35714	{0., 9.}	96.1851	1.89976	Reff	1.78676	1.45556	{1.01571, 1.869}	23.0664	ar	96920.	29669.5	{3., 75311.}	69.3876
5	R0	{2.26952, 9.62371}	{1.42337, 11.4263}	{{1.0158, 8.09734}, {2.20466, 17.2398}}	{38.8607, 38.2978}	inf	2.	1.12	{0., 3.}	79.2	1.04103	newly	5.	0.84	{0., 3.}	85.2	1.23228	Reff	2.26934	1.42334	{1.01578, 2.20448}	38.8579	ar	98881.	13218.	{1., 78222.}	86.6324
6	R0	{1.57191, 6.89004}	{1.57198, 15.4856}	{{1.11461, 10.2835}, {2.22641, 19.7105}}	{19.7946, 124.754}	inf	11.	2.31507	{0., 5.}	81.621	1.02454	newly	22.	1.19178	{0., 4.}	94.5828	1.72667	Reff	1.57147	1.58983	{1.18975, 2.22628}	19.8884	ar	95046.	28649.2	{1., 78841.}	69.8576
7	R0	{1.99226, 17.2339}	{2.22641, 14.8348}	{{2.22641, 14.8348}, {2.22641, 14.8348}}	{11.753, 13.9208}	inf	713.	1078.07	{84., 2587.}	79.8205	0.516974	newly	648.	669.707	{51., 1581.}	79.1693	0.512317	Reff	1.94486	2.18213	{2.11794, 2.22316}	12.1995	ar	97992.	84890.3	{84589., 85235.}	13.3702
8	R0	{2.63369, 15.1879}	{1.45278, 17.3097}	{{1.22395, 12.6353}, {1.75668, 19.7105}}	{44.8386, 22.387}	inf	35.	3.11111	{1., 6.}	94.0171	1.43299	newly	41.	1.71429	{0., 4.}	95.8188	1.71421	Reff	2.6309	1.47183	{1.30805, 1.86878}	44.056	ar	99451.	28235.4	{2., 75274.}	71.6087
9	R0	{2.53271, 9.90552}	{1.42337, 11.4263}	{{1.0158, 8.09734}, {2.20466, 17.2398}}	{44.0796, 37.2365}	inf	2.	1.13	{0., 3.}	70.5	0.673785	newly	8.	0.94	{0., 3.}	90.	1.37772	Reff	2.53258	1.42334	{1.01578, 2.20464}	44.0781	ar	99355.	14963.2	{1., 75209.}	84.9397
10	R0	{2.48791, 18.9003}	{1.91767, 15.3966}	{{1.30812, 12.378}, {2.64373, 19.5412}}	{25.5995, 19.6896}	inf	205.	72.5882	{4., 148.}	81.5522	1.05013	newly	173.	41.3922	{2., 84.}	80.6528	1.06159	Reff	2.47216	2.09542	{1.30795, 2.77634}	20.2088	ar	99291.	74983.5	{41735., 92434.}	24.481
11	R0	{2.84414, 18.4765}	{1.32172, 17.091}	{{1.18979, 13.6259}, {1.36512, 19.5412}}	{53.5284, 10.8682}	inf	68.	7.35135	{0., 19.}	92.8628	1.52443	newly	76.	3.32432	{0., 6.}	95.6259	1.7885	Reff	2.83774	1.34773	{1.18971, 1.66585}	52.5069	ar	99654.	27477.8	{6., 48788.}	72.4268
12	R0	{2.29369, 14.622}	{1.39974, 17.9813}	{{1.22395, 15.4344}, {1.66638, 19.7105}}	{38.9742, 26.4306}	inf	100.	5.71429	{1., 15.}	95.9473	1.60859	newly	114.	2.25714	{0., 5.}	98.0201	2.04805	Reff	2.28683	1.38931	{1.3079, 1.66605}	39.2474	ar	98982.	31502.	{5., 67563.}	68.174
13	R0	{2.73413, 16.7529}	{1.38646, 16.4564}	{{1.10133, 8.51468}, {1.66638, 19.7105}}	{49.2908, 18.4149}	inf	62.	6.12766	{0., 19.}	92.144	1.66894	newly	67.	3.59574	{0., 9.}	94.6332	1.88438	Reff	2.72932	1.44385	{1.10689, 1.79719}	47.0986	ar	99543.	26088.2	{2., 73196.}	73.792
14	R0	{1.79554, 16.5943}	{2.45965, 19.0916}	{{2.41419, 18.743}, {2.52006, 19.5955}}	{36.9869, 15.049}	inf	652.	1556.71	{291., 3526.}	104.155	0.383589	newly	541.	729.492	{130., 1648.}	84.8722	0.373414	Reff	1.75512	2.39631	{2.2747, 2.49282}	36.5323	ar	96821.	87275.5	{87852., 89631.}	9.85891
15	R0	{1.91429, 13.7771}	{1.94617, 14.0718}	{{1.94617, 14.0718}, {1.94617, 14.0718}}	{1.66537, 2.13906}	inf	266.	438.148	{76., 933.}	73.3021	0.391327	newly	278.	275.926	{44., 591.}	66.2137	0.408655	Reff	1.89691	1.92866	{1.90997, 1.94339}	1.6974	ar	97541.	76738.8	{77821., 78525.}	21.3267
16	R0	{1.42094, 10.5871}	{2.79994, 18.006}	{{2.46246, 15.904}, {2.86501, 18.4249}}	{97.0486, 70.0745}	inf	260.	885.695	{162., 2106.}	157.511	0.383153	newly	379.	490.356	{94., 1101.}	72.7427	0.297535	Reff	1.40736	2.76385	{2.4321, 2.85444}	96.3863	ar	93044.	92326.6	{88672., 93204.}	0.848405
17	R0	{1.45515, 6.16466}	{1.42337, 11.4263}	{{1.0158, 8.09734}, {2.20466, 17.2398}}	{26.9201, 88.8019}	inf	5.	1.74	{0., 4.}	66.	0.617399	newly	11.	1.55	{0., 4.}	87.1818	1.36356	Reff	1.45502	1.42333	{1.01577, 2.20442}	26.9197	ar	93254.	18141.6	{1., 75440.}	80.5461
18	R0	{2.85669, 13.2416}	{1.57218, 15.7074}	{{1.10133, 9.64612}, {2.12978, 19.7105}}	{44.9679, 28.6938}	inf	3.	2.32857	{0., 5.}	80.9091	1.00878	newly	8.	1.22857	{0., 3.}	84.6429	1.22417	Reff	2.85618	1.6008	{1.15957, 1.97174}	43.953	ar	99644.	29196.9	{1., 78582.}	70.6988
19	R0	{2.56417, 17.9558}	{1.26189, 15.2678}	{{1.18979, 13.6259}, {1.34361, 18.7523}}	{50.7877, 18.2139}	inf	91.	7.15789	{1., 20.}	95.382	1.66806	newly	91.	3.47368	{0., 8.}	96.1828	1.85421	Reff	2.55604	1.29078	{1.226, 1.36448}	49.5007	ar	99389.	24197.7	{5., 48531.}	75.6535
20	R0	{2.27517, 11.1864}	{1.53848, 14.9394}	{{1.0158, 8.09734}, {2.1968, 19.7105}}	{33.0457, 46.5089}	inf	8.	2.35714	{0., 5.}	85.2679	1.16429	newly	17.	1.37143	{0., 3.}	91.9328	1.53207	Reff	2.27446	1.57469	{1.11455, 1.97172}	31.4614	ar	98894.	23834.9	{1., 74748.}	75.8985
21	R0	{2.35735, 10.4735}	{1.45608, 11.8927}	{{1.0158, 8.09734}, {2.2194, 18.7523}}	{39.3633, 36.4416}	inf	5.	1.21	{0., 3.}	77.	0.938534	newly	5.	1.09	{0., 4.}	80.6	1.1242	Reff	2.35707	1.45605	{1.01578, 2.21938}	39.3583	ar	99036.	16688.5	{1., 73201.}	83.149
22	R0	{1.79551, 11.9179}	{2.54857, 14.6829}	{{2.48103, 13.7658}, {2.86501, 18.4249}}	{41.9415, 23.2002}	inf	143.	305.222	{13., 680.}	94.7683	0.542638	newly	205.	220.619	{7., 510.}	76.5931	0.559822	Reff	1.78616	2.53579	{2.44356, 2.85381}	41.9691	ar	97033.	86782.	{86226., 92907.}	10.5645
23	R0	{1.23545, 6.31687}	{2.74389, 16.9229}	{{2.48103, 13.7658}, {2.86501, 18.4249}}	{122.096, 167.9}	inf	66.	33.6462	{2., 89.}	74.3465	0.88562	newly	213.	20.6462	{1., 50.}	90.307	1.38152	Reff	1.23161	2.74777	{2.5254, 2.86487}	123.105	ar	88739.	87443.6	{88682., 93120.}	8.44419
24	R0	{2.24089, 10.1789}	{1.57085, 15.9113}	{{1.15964, 10.7075}, {1.97176, 19.7105}}	{30.7538, 59.1416}	inf	4.	2.36	{0., 5.}	66.	0.666789	newly	6.	1.10667	{0., 3.}	83.3333	1.18569	Reff	2.24062	1.5871	{1.18978, 1.97174}	30.0004	ar	98868.	36447.7	{1., 78439.}	63.1349
25	R0	{1.57298, 10.2607}	{1.57111, 17.2757}	{{1.34361, 13.4368}, {2.1968, 19.5412}}	{17.6318, 68.5101}	inf	107.	15.3889	{1., 29.}	89.7496	1.33749	newly	146.	7.16667	{0., 18.}	95.0913	1.71338	Reff	1.56721	1.59459	{1.34331, 2.21869}	17.8419	ar	94957.	43611.9	{5., 84701.}	54.072
26	R0	{1.2154, 8.2576}	{2.86701, 18.44}	{{2.86501, 18.4249}, {2.86501, 18.4249}}	{135.891, 123.309}	inf	374.	359.741	{27., 816.}	58.987	0.461749	newly	559.	194.138	{16., 415.}	65.8935	0.687782	Reff	1.19834	2.85032	{2.82905, 2.86386}	137.857	ar	88421.	93050.8	{92901., 93217.}	5.23614
27	R0	{2.22841, 18.7983}	{2.4257, 15.6891}	{{2.32014, 14.7828}, {2.57305, 16.884}}	{8.89097, 17.1743}	inf	579.	849.871	{63., 2330.}	83.9166	0.498687	newly	487.	523.065	{46., 1365.}	87.8386	0.481956	Reff	2.1875	2.40497	{2.21738, 2.56353}	9.95773	ar	98756.	88281.9	{86406., 90313.}	10.6061
28	R0	{1.27778, 5.36767}	{1.59498, 12.657}	{{1.0158, 8.09734}, {2.45321, 19.391}}	{36.2992, 138.489}	inf	15.	2.05	{0., 6.}	88.6111	1.48948	newly	37.	1.68	{0., 4.}	95.4595	1.96466	Reff	1.27717	1.59491	{1.01578, 2.45296}	36.3285	ar	89843.	22140.8	{1., 84032.}	75.4753
29	R0	{2.38042, 15.8901}	{1.43337, 16.7611}	{{1.18158, 11.0915}, {1.67375, 19.7105}}	{39.7852, 20.455}	inf	75.	6.25581	{0., 14.}	95.0351	1.78193	newly	93.	3.06977	{0., 7.}	96.6992	1.95748	Reff	2.37421	1.48728	{1.30785, 1.67325}	37.3569	ar	99112.	32639.8	{2., 68161.}	67.0678
30	R0	{2.53188, 12.7607}	{1.42337, 11.4263}	{{1.0158, 8.09734}, {2.20466, 17.2398}}	{44.0632, 32.2701}	inf	1.	1.18	{0., 3.}	78.	1.00723	newly	3.	0.89	{0., 3.}	77.6667	0.977718	Reff	2.5317	1.42334	{1.01577, 2.20459}	44.0606	ar	99317.	15130.2	{1., 74938.}	84.7658
31	R0	{1.24482, 10.5471}	{2.77354, 19.4633}	{{2.42074, 15.7308}, {2.81635, 19.898}}	{122.807, 84.5365}	inf	904.	2778.4	{297., 5971.}	153.621	0.419427	newly	1087.	1334.08	{146., 2791.}	78.2076	0.368929	Reff	1.19689	2.65026	{2.37621, 2.80142}	121.428	ar	89097.	92167.7	{88426., 92754.}	3.66638
32	R0	{2.30446, 17.9607}	{2.73016, 16.9769}	{{2.48103, 13.7658}, {2.86501, 18.4249}}	{18.7529, 9.04642}	inf	288.	113.865	{7., 258.}	76.4859	0.914844	newly	254.	68.0946	{4., 155.}	76.1811	0.934748	Reff	2.28427	2.74185	{2.47344, 2.86444}	20.242	ar	99028.	90632.9	{88845., 93187.}	8.47755
33	R0	{2.31579, 11.8236}	{1.53745, 12.4416}	{{1.0158, 8.09734}, {2.22832, 19.391}}	{35.7286, 32.5047}	inf	22.	2.79	{0., 8.}	90.0357	1.69032	newly	33.	1.87	{0., 6.}	94.3333	1.88377	Reff	2.31442	1.53735	{1.01576, 2.2283}	35.7016	ar	99012.	21179.7	{1., 84688.}	78.609
34	R0	{1.6678, 10.6744}	{2.71473, 17.5734}	{{2.32014, 14.7828}, {2.86501, 18.4249}}	{62.7733, 64.6315}	inf	174.	99.0794	{8., 256.}	67.9654	0.795496	newly	267.	56.8095	{3., 156.}	78.9133	1.05086	Reff	1.65861	2.72907	{2.31877, 2.86361}	64.5398	ar	95809.	85785.9	{86371., 93177.}	10.4616
35	R0	{1.65476, 7.07559}	{1.45608, 11.8927}	{{1.0158, 8.09734}, {2.2194, 18.7523}}	{26.8495, 71.9444}	inf	1.	1.56	{0., 3.}	83.5	1.10896	newly	10.	1.65	{0., 3.}	95.5	1.44019	Reff	1.65458	1.45605	{1.01577, 2.21933}	26.8477	ar	95746.	15026.5	{1., 72456.}	84.3059
36	R0	{2.71807, 18.5959}	{1.37361, 18.1144}	{{1.22395, 15.6333}, {1.56495, 19.5412}}	{49.4639, 5.87727}	inf	74.	5.29412	{1., 13.}	94.0516	1.45298	newly	61.	2.2549	{0., 5.}	96.3034	1.8011	Reff	2.71258	1.39922	{1.34318, 1.66616}	48.4174	ar	99540.	35448.3	{5., 66730.}	64.3879
37	R0	{2.6202, 12.7552}	{1.42825, 17.4315}	{{1.13239, 13.8982}, {1.75668, 19.7105}}	{45.4906, 38.9044}	inf	2.	3.60784	{1., 7.}	55.7423	0.493415	newly	7.	1.52941	{0., 4.}	80.9524	1.11602	Reff	2.61991	1.44338	{1.18133, 1.75661}	44.9072	ar	99426.	30357.2	{2., 71339.}	69.4675
38	R0	{1.47672, 9.81651}	{2.60307, 16.974}	{{2.32014, 14.7828}, {2.86656, 18.9813}}	{76.2735, 72.9125}	inf	196.	241.241	{9., 566.}	59.6386	0.54991	newly	329.	136.603	{4., 320.}	60.4287	0.693598	Reff	1.46491	2.58756	{2.29292, 2.86094}	76.6367	ar	93815.	88477.7	{86253., 93122.}	5.68913
39	R0	{1.90004, 12.3313}	{1.51572, 15.9726}	{{1.20956, 12.378}, {1.91377, 19.7105}}	{22.0827, 33.1736}	inf	121.	10.5192	{0., 24.}	93.8842	1.78465	newly	167.	5.90385	{0., 12.}	96.4648	2.15219	Reff	1.89271	1.5305	{1.20952, 1.91333}	21.3625	ar	97413.	38556.8	{4., 76985.}	60.4193
40	R0	{2.64218, 18.5237}	{2.32741, 17.1681}	{{1.34361, 12.6353}, {2.86501, 18.7523}}	{21.0432, 8.32259}	inf	208.	61.625	{3., 163.}	79.3683	1.17036	newly	187.	33.7344	{1., 89.}	81.9936	1.25854	Reff	2.62693	2.38963	{1.34346, 2.86406}	19.3797	ar	99474.	73652.7	{12., 93151.}	25.9578
41	R0	{1.6945, 7.17996}	{1.45608, 11.8927}	{{1.0158, 8.09734}, {2.2194, 18.7523}}	{27.331, 69.5903}	inf	3.	1.2	{0., 3.}	76.8	0.953763	newly	9.	0.87	{0., 3.}	90.3333	1.4187	Reff	1.69433	1.45605	{1.01578, 2.21933}	27.3292	ar	96332.	18015.9	{1., 73733.}	81.2981
42	R0	{1.84559, 18.0074}	{2.77291, 19.53}	{{2.76795, 19.6041}, {2.81635, 19.898}}	{50.2453, 9.97261}	inf	1046.	1527.03	{212., 3492.}	68.9891	0.41737	newly	870.	765.06	{101., 1699.}	65.042	0.427999	Reff	1.78061	2.6941	{2.57907, 2.8003}	51.3024	ar	97358.	92034.4	{91849., 92700.}	5.46808
43	R0	{2.24965, 17.0361}	{2.79606, 18.0169}	{{2.32014, 14.7828}, {2.86501, 18.4249}}	{24.6478, 9.20175}	inf	172.	208.266	{13., 407.}	65.7735	0.521721	newly	161.	116.844	{7., 225.}	62.9076	0.572403	Reff	2.23721	2.78488	{2.32, 2.86406}	24.5017	ar	98770.	90833.1	{86691., 93160.}	8.03575
44	R0	{2.54324, 15.7967}	{1.34217, 17.1415}	{{1.04117, 8.84894}, {1.66638, 19.5412}}	{47.2259, 21.6771}	inf	68.	3.66667	{0., 8.}	96.8661	1.73342	newly	107.	1.76923	{0., 5.}	98.3465	2.22919	Reff	2.53714	1.39968	{1.04111, 1.66633}	44.8325	ar	99354.	29605.1	{2., 67955.}	70.2024
45	R0	{2.54943, 14.8032}	{1.43084, 16.3939}	{{1.10133, 10.3394}, {1.67375, 19.7105}}	{43.8761, 24.2385}	inf	40.	2.50847	{0., 5.}	95.5992	1.61211	newly	50.	1.42373	{0., 3.}	97.1525	1.91415	Reff	2.54637	1.46925	{1.1463, 1.75664}	42.3001	ar	99380.	22107.1	{1., 68075.}	77.755
46	R0	{2.37755, 12.6461}	{1.42337, 11.4263}	{{1.0158, 8.09734}, {2.20466, 17.2398}}	{41.0287, 32.3451}	inf	8.	2.86	{0., 11.}	83.625	1.50605	newly	26.	1.99	{0., 6.}	92.5	1.8832	Reff	2.37696	1.42327	{1.01578, 2.20464}	41.0193	ar	99096.	20307.2	{1., 78516.}	79.5076
47	R0	{1.69279, 11.6636}	{2.19848, 14.9526}	{{1.86902, 14.8348}, {2.22641, 14.8348}}	{30.4036, 28.6924}	inf	138.	107.333	{10., 196.}	63.3663	0.622157	newly	167.	68.9216	{9., 125.}	66.7606	0.697049	Reff	1.68417	2.19929	{2.20481, 2.22592}	30.614	ar	96214.	82663.7	{84466., 85189.}	14.0835
48	R0	{1.82402, 10.0038}	{1.43602, 16.3948}	{{1.11164, 8.84894}, {1.67375, 19.7105}}	{22.5496, 68.824}	inf	25.	4.23438	{0., 9.}	87.1686	1.25741	newly	39.	1.95313	{0., 5.}	94.992	1.75844	Reff	1.82252	1.4616	{1.29987, 1.75649}	20.8868	ar	97076.	34403.	{2., 67688.}	64.5607
49	R0	{1.6424, 11.1667}	{2.90125, 15.001}	{{2.44675, 11.6528}, {2.99911, 15.963}}	{76.6467, 35.8045}	inf	267.	670.569	{78., 1619.}	117.216	0.40922	newly	380.	501.722	{60., 1156.}	90.3363	0.403198	Reff	1.62686	2.86774	{2.44359, 2.9926}	76.2741	ar	95546.	92877.3	{88548., 94151.}	2.7931
50	R0	{2.05638, 14.3048}	{2.35798, 15.6935}	{{1.34361, 11.6151}, {2.86501, 18.4249}}	{28.9698, 21.9395}	inf	167.	54.75	{1., 159.}	75.2262	0.981958	newly	176.	33.4808	{1., 95.}	80.9768	1.07536	Reff	2.0461	2.42264	{1.51157, 2.86412}	30.5976	ar	98194.	78382.7	{47801., 93175.}	20.1757
